# Melanocytic lesions ≤ 6mm: Prospective series of 481 melanocytic trunk and limb lesions in Brazil

**DOI:** 10.1371/journal.pone.0252162

**Published:** 2021-06-08

**Authors:** Gabriella Campos-do-Carmo, Aretha Brito Nobre, Tullia Cuzzi, Giuseppe Argenziano, Carlos Gil Ferreira, Luiz Claudio Santos Thuler

**Affiliations:** 1 Gávea Medical Center, Rio de Janeiro, Brazil; 2 National Cancer Institute, Rio de Janeiro, Brazil; 3 Federal University, Rio de Janeiro, Brazil; 4 Dermatology Unit, University of Campania, Caserta, Italy; 5 Oncoclinicas Institute for Research and Education, Rio de Janeiro, Brazil; University of Queensland Diamantina Institute, AUSTRALIA

## Abstract

Early diagnosis when melanoma is still small and thin is essential for improving mortality and morbidity. However, the diagnosis of small size melanoma might be particularly difficult, not only clinically but also dermoscopically. This study aimed to define clinical and dermatoscopic parameters in the diagnosis of suspicious pigmented cutaneous lesions with a diameter of ≤ 6mm and determine the sensitivity, specificity, positive and negative predictive values as well as the accuracy of each clinical and dermatoscopic criterion. This is a transversal, descriptive and analytical study of dermatoscopic analysis with the gold standard being the pathologic examination obtained from the excisional biopsy of suspicious melanocytic lesions with a diameter of ≤ 6mm. Trunk and limb lesion data from a public health service and a private clinic were prospectively collected from 2011 to 2017 by a unique observer. In total, 481 melanocytic lesions were included, with 73.8% being ≤ 4mm in diameter. Overall, 123 were diagnosed as melanoma, 56.0% in situ and 22.0% as thin melanomas (Breslow index 0.1 to 1.0mm). Melanoma presented symmetry in 53.7% of cases, regular borders in 54.5% and a single color in 60.2%. Regarding evolution, 13.8% of melanomas versus 10.9% of benign lesions (p = 0.116) were new by comparing photos from baseline with photos from the follow-up. The majority of melanomas (65%) were found on the limbs compared to 37.2% of the benign lesions at this location (p<0.001). A multiple logistic regression model adjusted for age, gender and location was created. The independent variables associated with the diagnosis of melanoma ≤ 6mm, adjusted for age, gender and location, were: streaks (adjusted Odds Ratio [aOR] 2.5; 95% CI 1.3–4.7; p = 0.006), and the presence of a structureless area (aOR 2.2, 95% CI 1.2–4.0, p = 0.011). Conversely, a symmetric typical pigment network was a protection variable (aOR 0.4, 95% 0.7–0.9, p = 0.040). In conclusion, dermatoscopic criteria have been identified which help to diagnose cases of small size melanoma. These include streaks and structureless areas that can be taken, particularly in consideration for the diagnosis of this subset of small difficult melanomas.

## Introduction

Cutaneous melanoma (CM) is the cancer with the highest mortality, despite representing only 1% of all skin cancers [1]. Its metastatic potential, its increasing incidence in white populations and its prevalence in lower age groups has stimulated ever more early diagnoses to improve its prognosis [2]. To assist in the early diagnosis, dermatologists currently rely on dermoscopy. At present, this is the most important tool to assess pigmented cutaneous lesions and allow the early excision and treatment of the CM in order to avoid the unnecessary removal of benign lesions. With the advent of the dermatoscope and routine examination of pigmented lesions, the number of melanomas in initial stages with sizes under the classic 6mm in diameter criterion by ABCD for CM are increasingly being detected [3–16]. This new dimensional frontier for melanomas has been reported in the literature in case reports as well as in case series, predominantly comparing them with larger melanomas, with many articles using the histopathological dimensions of the lesions [17–24].

However, what distinguishes these small lesions from the melanocytic nevi? Does dermoscopy help in this distinction? Are dermatoscopic criteria for large melanomas applicable to those of smaller size? It is therefore essential to better characterize small suspicious melanocytic lesions, allowing the ideal moment for their excision to be established. This study aimed to define clinical and dermatoscopic parameters in the diagnosis of suspicious pigmented cutaneous lesions with a diameter of ≤ 6mm and determine the sensitivity, specificity, positive and negative predictive values as well as the accuracy of each clinical and dermatoscopic criterion.

## Materials and methods

This is a clinical-epidemiological study with transversal, descriptive and analytical design using data from both public health service (Dermatologic Department of the Brazilian National Cancer Institute–INCA), as well as from private clinic, where the leading researcher (Campos-do-Carmo G), carries out the same dermoscopy activity and receives referred patients. Clinical and dermatoscopic images obtained from 2011 to 2017 were prospectively collected from 521 small-diameter pigmented lesions, according to the following inclusion criteria: Trunk and limb pigmented lesions of ≤ 6mm in diameter suspicious of cutaneous melanoma either at clinical examination or dermoscopy, submitted to biopsy; patient consent to take part in the study by signing the Informed Consent Form.

Lesions included had to meet the dermatoscopic melanocytic hypothesis and a suspicion of melanoma in at least one of two analyses (clinical and/or dermatoscopic). The criteria for exclusion were: suspicious clinical lesion but not melanocytic at dermoscopy, rather typical of other lesions as angiomas, seborrheic keratosis and basal cell carcinoma; lesions on the face, scalp, acral and mucosal surfaces; metastatic lesions; insufficient or poor quality photos; and technical problems during the inclusion of small lesions in paraffin. Forty lesions were excluded by the criteria above. All lesions were measured *in vivo* before excision, using a millimeter scale tag attached to the relaxed skin.

The lesions were photographed using digital cameras (Nikon, Tokyo, Japan) and dermoscopy images were made using a coupler and manual dermatoscope 10X, (DermLite® Foto; 3GEN, USA), and/or digital Mediscope 20X (Fotofinder®, Germany). Image acquisition was performed under crossed polarized light with alcohol gel as the interface liquid. The decision to remove the lesion was based on clinical and/or dermatoscopic suspicion at the time of examination. The clinical hypothesis was based on the ABCDE criteria (asymmetry, irregular border, colour, diameter and evolution) proposed by Friedman *et al*. in 1985 [25]. The dermatoscopic hypothesis represented the presence of certain dermatoscopic structures and compared the clinical and dermatologic hypotheses with the final histological result. The protocol for monitoring patient’s evolution was based on total body photos once a year and a revision of lesions at high clinical-dermoscopic suspicion for melanoma.

The leading researcher in charge of patient evaluation already had over 10 years of experience in dermoscopy at the time of study onset. After selection and lesion removal, in a secondary phase, the cataloged clinical and dermatoscopic images were displayed on the computer screen and submitted to the simplified ABC dermatoscopic algorithm, without access to histopathological results at that time, in order to quantify the variables being analyzed in relation to the algorithm, with the results found by histopathology. The cutoff point for the simplified ABC dermoscopic algorithm was defined according to the literature (≥ 3mm) [26] and according to the data distribution characteristics (≥ 4mm). Demographic data were taken from the questionnaire prepared by the researcher both from INCA patients and private clinic patients’ records.

Histopathological reports were consulted from the INCA database or collected with patients and pathologists. All lesions were examined by pathologists with over 5 years of experience in melanocytic lesions, and project collaborators. The dependent variable was the CM diagnosis from the histopathological examination. The descriptive and independent variables were sociodemographic, personal and family information regarding melanoma and melanocytic nevi, clinical lesion features, clinical hypothesis, dermatoscopic features and dermatoscopic hypothesis of the lesion.

### Statistical analysis

The descriptive analysis of the lesions of the study considered the mean (± standard deviation) or median for continuous variables and frequency distribution for categorical variables. Non-parametric tests were applied to analyze results considering the nature of distribution of the studied variables. The chi-squared test was employed aiming to compare the frequency of categorical variables and, when indicated, Fisher’s Exact Test was applied. The association between independent variables and the outcome (melanoma diagnosis) was made by applying the Pearson’s chi-square test. Values of p<0.05 were considered statistically significant.

The *Statistical Package for the Social Sciences* (SPSS) for Windows, Inc., USA, version 21.0 was used for statistical calculations in this study. Calculations of Sensitivity (S), Specificity (E), Positive Predictive Value (PPV), Negative Predictive Value (NPV) and Accuracy were performed using the online software MedCalc, available at: https://www.medcalc.org/calc/diagnostic_test.php.

The procedures utilized in this research were in accordance with all resolutions that regulate research directives and research norms involving humans in Brazil. This project was approved by the Committee on Ethics and Research from INCA, under Number 130–10 on Feb 16, 2011 (45756015.3.0000.5274) and amendment, Number 1.295.179 of October 25, 2015. All patients consented and signed the consent form.

## Results

The analysis included 481 lesions with diameters not exceeding 6mm and which were suspected of cutaneous melanomas, resulting in the detection of 123 CMs (Table 1). The lesions were found in a total of 372 patients, with a median of 1 lesion per patient. All excised lesions were confirmed as melanocytic by histopathology.

**Table 1:** Histopathological results of 481 lesions ≤ 6mm in diameter.

Histopathologic Results		N	%
Atypical melanocytic nevus		217	45.1
Cutaneous melanoma		123	25.6
*In situ*	69 (56.0)		
Breslow thickness ≤ 1mm	27 (22.0)		
Atypical melanocytic proliferations/Incipient melanomas	27 (22.0)		
Common melanocytic nevi		74	15.4
Lentigo simplex		36	7.5
Junctional melanocytic lentiginous nevus		16	3.3
Spitz Nevus/Reed Nevus		10	2.1
Halo nevus		4	0.8
Blue nevus		1	0.2
Total		481	100.0

Lesion sizes varied from 1 to 6mm. Almost half of the documented lesions (48.2%) had diameters of up to 3mm, 73.8% up to 4mm and 91.7% up to 5mm. Of the CMs found, 50.4% had diameters ≤ 3mm and 75.6% ≤ 4mm. The remaining 24.4% had diameters between 5 and 6mm.

The majority of CMs were examined *in situ* (56.0%). The most prevalent histological subtype was superficial spreading melanoma (94 cases), with only one case associated with melanocytic nevus, one case of spitzoid melanoma and 27 non-classified incipient melanomas. The Breslow thickness index varied from 0.1 to 1.0mm, with a median diameter of 0.3mm.

The analysis included 226 lesions from patients seen at the public hospital and 255 from private clinics. Patients were similar regarding distribution by sex, lesion diameter and evolution. However, there were differences in age, personal history and family history regarding CM, reflecting the profiles of the patients that use the two locations of the study in Brazil.

The mean age at the time of diagnosis was higher among patients with CM (52.5±14.2) than without CM (46.9±15.5; p<0.001). The age of most patients, with or without CM, ranged between 31 and 50 years (S1 Table).

Family history regarding CM was statistically more prevalent in the group with CM (37.4% versus 26.5%; p = 0.022), with an average of 0.54 (±0.84) affected family members in CM versus 0.32 (±0.61) in non-CM (p = 0.003).

The analyzed lesions were predominantly flat or macular (S2 Table), with differences between CM and benign cases (87.0% versus 74.9%, p = 0.005). Regarding location, most CM cases were found on the limbs rather than the trunk (65.0% versus 37.4%; p<0.001).

The atypical and asymmetrical pigmentary network occurred in 62.6% cases of CM and in 54.7% of non-CM cases, while the atypical and symmetrical pigmentary network occurred in 5.7% of CM and in 13.7% of benign lesions with p = 0.049 (Table 2).

**Table 2:** Dermatoscopic characteristics of 481 melanocytic lesions ≤ 6mm in diameter.

Variables	CM N (%)	Non-CM N (%)	p
Regular pigmentary network			0.197
Symmetrical	9 (7.3)	48 (13.4)	
Asymmetrical	52 (42.3)	141 (39.4)	
No	62 (50.4)	169 (47.2)	
Atypical pigmentary network			**0.049**
Symmetrical	7 (5.7)	49 (13.7)	
Asymmetrical	77 (62.6)	196 (54.7)	
No	39 (31.7)	113 (31.6)	
Negative network			0.816
Yes	2 (1.6)	7 (2.0)	
No	121 (98.4)	351 (98)	
Streaks			**0.002**
Yes	25 (20.3)	34 (9.5)	
No	98 (79.7)	324 (90.5)	
Globules			0.244
Symmetrical	5 (4.1)	31 (8.7)	
Asymmetrical	57 (46.3)	155 (43.3)	
No	61 (49.6)	172 (48.0)	
Dots			0.154
Symmetrical	4 (3.3)	30 (8.4)	
Asymmetrical	75 (61.0)	202 (56.4)	
No	44 (35.8)	126 (35.2)	
Structureless area			**0.006**
Yes	106 (86.2)	265 (74.0)	
No	17 (13.8)	93 (26.0)	
Blue-whitish veil			0.110
Yes	4 (3.3)	4 (1.1)	
No	119 (96.7)	354 (98.9)	
Peppering			0.546
Yes	25 (20.3)	64 (17.9)	
No	98 (79.7)	294 (82.1)	
Chrysalis			0.435
Yes	3 (2.4)	5 (1.4)	
No	120 (97.6)	353 (98.6)	
Brown color			0.863
Yes	104 (84.6)	305 (85.2)	
No	19 (15.4)	53 (14.8)	
Black color			0.334
Yes	24 (19.5)	85 (23.7)	
No	99 (80.5)	273 (76.3)	
Gray-bluish color			0.771
Yes	61 (49.6)	183 (51.1)	
No	62 (50.4)	175 (48.9)	
White color			**0.023**
Yes	3 (2.4)	1 (0.3)	
No	120 (97.6)	357 (99.7)	
Red color			0.075
Yes	14 (11.4)	23 (6.4)	
No	109 (88.6)	335 (93.6)	

Statistically significant p values are shown in bold type. CM = Cutaneous Melanoma.

Streaks were more prevalent in CM cases compared to benign cases (20.3% versus 9.5%; p = 0.002). Structureless areas prevailed in CM with 86.2% of cases versus 74.0% in benign lesions (p = 0.006). Regarding colors, white was only present in 3 CM cases and 1 non-CM case (2.4% versus 0.3%; p = 0.023).

There were clinical hypotheses of CM in only 36.6% of the confirmed cases. Therefore, 63.4% of the CMs would not have been diagnosed without dermoscopy. On the other hand, 92.7% of 123 melanomas had a dermatoscopic hypothesis of CM. Also, 29.3% of confirmed CM cases had both a clinical and dermatoscopic hypothesis of CM. The excision of 32.4% of melanocytic nevi was carried out due to clinical suspicion and 88.8% of nevi were excised to rule out CM at dermoscopy (Table 3).

**Table 3:** Comparison between clinical and dermatological hypotheses of cutaneous melanoma of 481 analyzed melanocytic lesions ≤ 6mm in diameter.

Variables	CM N (%)	Non-CM N (%)
Clinical hypothesis		
CM	45 (36.6)	116 (32.4)
Other hypothesis	78 (63.4)	242 (67.6)
Dermatoscopic hypothesis		
CM	114 (92.7)	318 (88.8)
Other hypothesis	9 (7.3)	40 (11.2)
Clinical hypothesis and dermatoscopic hypothesis		
CM (both clinical and dermatoscopic)	36 (29.3)	76 (21.2)
Clinical or dermatoscopic hypothesis	87 (70.7)	282 (78.8)

CM = Cutaneous Melanoma.

The clinical and dermatoscopic criteria were analyzed according to their sensitivity, specificity, positive predictive value, negative predictive value and accuracy (Table 4).

**Table 4:** Validity of clinical and dermatoscopic criteria of 481 lesions ≤ 6mm in diameter.

Variables	Frequency N (%)	Se %	Sp %	PPV %	NPV %	Accuracy %
**Clinical CM hypothesis**	161 (33.5)	36.6	67.6	27.9	75.6	59.7
Asymmetry	214 (44.5)	46.3	56.1	26.6	75.3	53.6
Irregular borders	208 (43.2)	45.5	57.5	26.9	75.5	54.5
Colors (more than one)	182 (38.0)	39.8	62.8	26.9	75.2	57.0
Evolution: new or changed	195 (40.5)	42.3	60.1	26.7	75.2	55.5
Macula	375 (78.0)	87.0	25.1	28.5	84.9	41.0
**Dermatoscopic CM hypothesis**	432 (90.0)	92.7	11.2	26.4	81.6	32.0
Regular symmetrical pigmentary network	57 (12.0)	7.3	86.6	15.8	73.1	66.3
Regular asymmetrical pigmentary network	193 (40.0)	42.3	60.6	26.9	75.3	55.9
Atypical symmetrical pigmentary network	56 (12.0)	5.7	86.3	12.5	72.7	65.7
Atypical asymmetrical pigmentary network	273 (57.0)	62.6	45.2	28.2	77.9	49.7
Negative network	9 (2.0)	1.6	98.0	22.2	74.4	73.4
Symmetrical streaks	26 (5.4)	11.4	94.7	42.4	75.7	73.4
Asymmetric streaks	33 (6.9)	11.4	75.7	11.4	94.7	73.4
Symmetrical globules	36 (7.5)	4.1	91.3	13.9	73.5	69.0
Asymmetric globules	212 (44.1)	46.3	56.7	26.9	75.5	54.0
Symmetrical dots	34 (7.1)	3.2	91.6	11.7	73.4	69.0
Asymmetric dots	278 (58.0)	61.0	43.3	25.6	27.0	47.8
Structureless area	371 (77.0)	86.2	26.0	28.6	84.5	41.4
Blue-whitish veil	8 (2.0)	3.2	98.9	50.0	74.9	74.4
Peppering	89 (18.0)	20.3	82.1	28.1	75.0	66.3
Chrysalis	8 (2.0)	2.4	98.6	37.5	74.6	74.0
Brown color	409 (85.0)	84.5	14.8	25.4	73.6	32.6
Black color	109 (23.0)	19.5	76.3	22.0	73.4	61.7
Gray-bluish color	244 (51.0)	49.6	48.9	25.0	73.4	49.0
White color	4 (1.0)	2.4	99.7	75.0	74.8	74.8
Red color	37 (8.0)	11.4	93.6	37.8	75.4	72.6
**Clinical and dermatoscopic CM hypothesis**	112 (23.3)	29.3	78.8	32.1	76.4	66.1
Final score ≥ 4 of the simplified ABC dermatoscopic algorithm	261(54.3)	61.8	48.3	29.1	78.6	51.8
Final score ≥ 3 of the simplified ABC dermatoscopic algorithm	384 (79.8)	86.2	22.3	27.6	82.5	38.7

CM = Cutaneous Melanoma; Se = Sensibility; Sp = Specificity; PPV = Positive Predictive Value; NPV = Negative Predictive Value.

The clinical CM hypothesis was present in only 33.5% of the lesions, with 36.6% sensitivity and 67.6% specificity. Asymmetry was the clinical criteria with greatest sensitivity (46.3%) of ABCDE, but the presence of more than one color had the highest clinical specificity for melanoma (62.8%) and highest accuracy (57%). Clinically irregular borders were only found in 43.2% of the pigmented lesions studied, with the presence of the clinical evolution criterion (new or modified lesion) in 40.5%. The elementary macula lesion corresponded to the majority of lesions (78.0%), with a sensitivity of 87.0%, specificity of 25.0% and accuracy of 41.0%.

**Fig 1:**
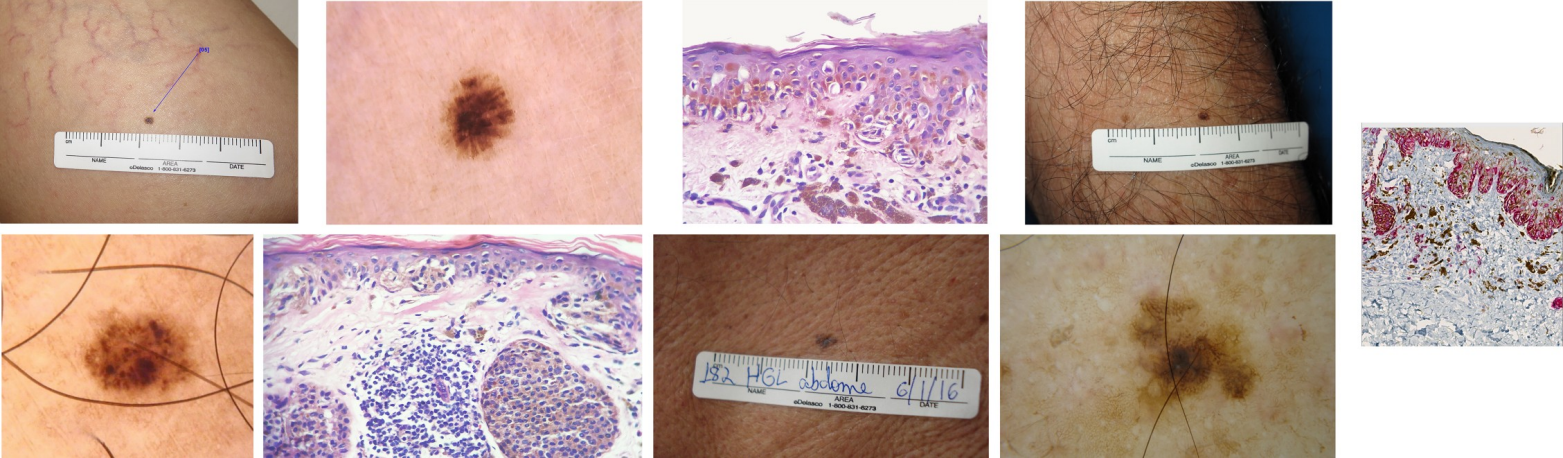
Case **1:** Fig 1A: Clinically non-suspected lesion; Fig 1B: Suspected dermoscopy lesion with asymmetric structureless area; Fig 1C: H&E (Hematoxylin & Eosin 40X) Melanocytic lentiginous proliferation with small junctional nests. Some melanocytes adopt a supra-basal position. In the dermis, mononuclear inflammatory infiltrates include melanophages. Conclusion: 2mm diameter melanoma, Breslow 0.2mm. Case **2:** Fig 1D: Clinically suspected lesion. Fig 1E: Suspected dermoscopy lesion with asymmetric globules; Fig 1F: H&E (40X) Melanocytes with hyperchromatic nucleus, enlarged, and moderately pleomorphic. They form nests of varied size where they are eventually horizontal. Intradermal nests consist of cells with mild pleomorphism adjacent to predominantly lymphocytic inflammatory infiltrates. Conclusion: 3mm diameter melanoma, Breslow 0.4mm. Case **3:** Fig 1G: Clinically suspected lesion. Fig 1H: Suspected dermoscopy lesion with atypical asymmetrical pigmentary network. Fig 1I: Immunohistochemistry with Melan A (10X), showing the junctional melanocytic proliferation, the migration of melanocytes to the layers of the epidermis and the presence of Melan A positive cells in the dermis (loose), amid the mononuclear inflammatory infiltrate with melanophages. Conclusion: 5mm diameter melanoma, Breslow 0.2mm.

**Fig 2:**
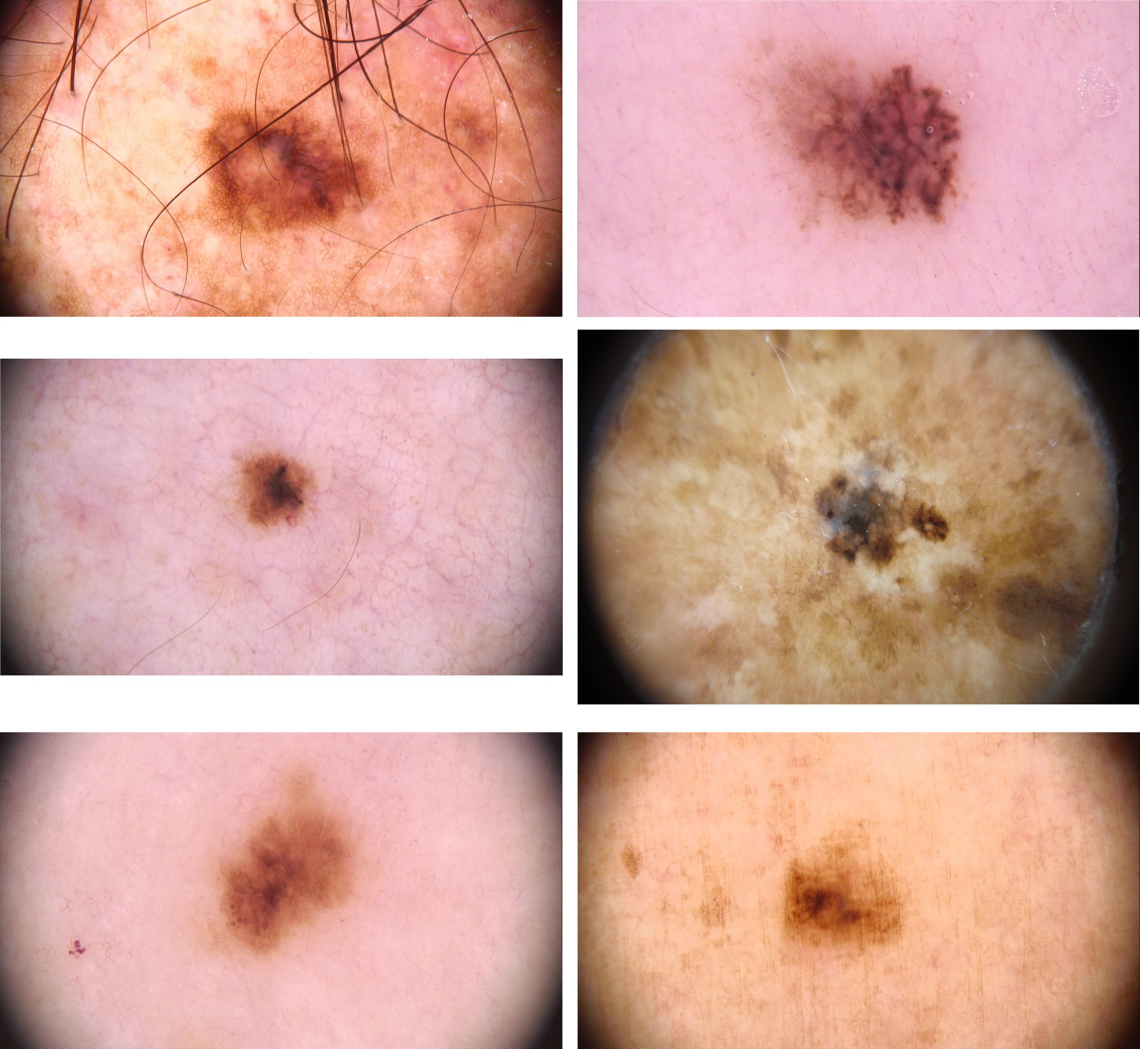
Dermoscopic structures that helped with the diagnosis of some small size melanomas. Fig 2A –Asymmetrical pigmentary network, asymmetric structureless area, blue-whitish veil. Fig 2B –Asymmetrical structureless area and streaks. Fig 2C –Asymmetrical structureless area, asymmetrical globules. Fig 2D –Asymmetrical pigmentary network and streaks. Fig 2E –Blue-whitish veil, asymmetric; asymmetrical pigmentary network. Fig 2F –Asymmetrical pigmentary network and asymmetrical structureless area.

The dermatoscopic CM hypothesis was present in 90.0% of lesions with 92.7% sensitivity, 11.2% specificity and 81.6% NPV. The dermatoscopic criteria with highest sensitivity were: structureless area (86.2%), brown color (84.5%), atypical asymmetric pigmentary network (62.6%) and asymmetric dots (61.0%). The highest specificities of the dermatoscopic structures analyzed were: white color (99.7%), blue-whitish veil (98.9%), chrysalis (98.6%), and negative network (98.0%). The highest accuracies were for white color (74.8%), blue-whitish veil (74.4%), chrysalis (74.0%) negative network (73.4%), asymmetric streaks (73.4%) and symmetrical streaks (73.4%) (Figs 1A–1I and 2A–2F).

The following variables were included in the multiple logistic regression model, which in the univariate analysis presented p<0.20 (Tables 2, S1 and S2): the presence of streaks (0.002), macula (0.005), structureless area (0.006), family history of melanoma (0.022), white color (0.023), symmetrical atypical pigment network (0.049), red color (0.075), atypical nevi (0.147), clinical evolution (0.116), blue-whitish veil (0.110), size in millimeters (0.154), dots (0.154), eye color (0.183), and regular pigmentary network (0.197).

In the multivariate analysis, the following variables remained in the adjusted model: streaks (adjusted Odds Ratio [aOR] 2.5; 95% CI 1.3–4.7; p = 0.006), and the presence of structureless area (aOR 2.2, 95% CI 1.2–4.0, p = 0.011). Conversely, a symmetric typical pigment network was a protection variable (aOR 0.4, 95% 0.7–0.9, p = 0.040). Multivariate analysis was adjusted by age (continuous), gender, study site, and limb localization.

## Discussion

In the present study, the melanoma dimensions ranged from 2 to 6 mm. Although ≤ 6mm is not really very small, this cutoff point was used because the clinical ABCD considers that a lesion with a diameter > 6mm must be considered suspicious of CM. [12]. Dermoscopy was performed in all cases. The dermatoscopic hypothesis had a sensitivity of 92.7%, but a low specificity of 11.2%. The accuracy of the clinical hypothesis (59.7%) and that of the dermatoscopic hypothesis (32%), separately, increased to 66.1%, when both hypotheses were associated. These results are in accordance with Grichnik [5], who suggests that dermoscopy should be considered as auxiliary to the clinical examination, but there are cases where dermoscopy alone is insufficient for a diagnosis of precocious melanomas, with the diagnostic accuracy increased by accessing the lesion dermoscopy in issue and comparing it with the remaining patient’s nevi (“ugly duck” sign). We disagree with Carli *et al*. [27], who reported that joining the clinical and dermatoscopic hypotheses did not help with the diagnosis of small melanocytic lesions, only with large and intermediate lesions. Many of the reported cases are similar to those reported by Salerni *et al*. [28], who reported 8 melanomas, with an average of 3.7mm in diameter, 7 of which were in the lower limbs, without suspicious clinical criterion, found through the routine use of dermoscopy in all lesions and monitoring high risk patients with digital dermoscopy.

The present study did not compare small melanomas with those of sizes exceeding 6mm, as in the study by Seidenari *et al*. [16]. Nevertheless, we confirm that some dermatoscopic criteria of high suspicion in melanomas exceeding 6mm, such as the gray-bluish veil, chrysalis and negative network, despite having high specificity in our casuistic (98.9, 98.6 and 98.0%), presented low sensitivity. On the other hand, the highest sensitivities were found in the following structures: brown color (84.5%), structureless area (86.2%), atypical asymmetric pigmentary network (62.6%), and asymmetric dots (61.0%), with corresponding accuracies of 32.6%, 41.4%, 49.7%, and 47.8%, respectively.

In our study of lesions ≤ 6 mm, most were macules (78.0%) and 56% of the melanomas were in situ. The structureless area was a dermoscopic criterion with a sensitivity of 86.2% and negative predictive value of 84.5% (p = 0.006) that was retained for use in the multivariate logistic regression model. Its presence in small melanomas and those in situ caught the attention of our study, as it did with Annessi et al. (2007), who found that areas without a light brown structure were the highest statistically significant discriminator and main predictor of thin melanoma (positive precictive value of 93.8%) after studying 198 atypical melanocytic macular lesions > 5 mm [29]. In a study of dermoscopic criteria that did not analyse lesion size, when compared with nevi the criteria that remained powerful indicators of in situ melanomas were irregular hyperpigmented areas and prominent skin marks [30].

Our study used the modified ABC-point list algorithm proposed by Blum, Rassner and Garbe (2003), but in an unprecedented way, analysing melanocytic lesions ≤ 6 mm. It is a very simple algorithm and obtained high sensitivity, specificity and diagnostic accuracy compared to the ABCD rule, Menzies score, seven-point checklist and seven features for melanoma [26].

More recently, a multicentric study designed a scoring classifier diagnostic method, the iDScore by combining clinical data of the patient with dermoscopic features of the melanocytic lesions. The accuracy of the iDScore in differentiating early melanoma from atypical nevi in a teledermoscopy setting was tested and compared it with intuitive diagnosis, the ABCD rule and the seven-point checklist. The platform designed for the iDScore project provides ready support for physicians of any dermoscopy skill level and is useful for education and training [31].

In this study we observed a high percentage of melanomas in the limbs, those areas particularly subject to photoexposure. In a recent study that combined the anatomical location of atypical melanocytic skin lesions into an algorithm for discriminating early melanomas from atypical nevi, the largest proportion of correctly identified early melanomas (41.5%) was in the chronically photoexposed areas [31].

In the present study, the criterion evolution (new or modified) found in 40.5% of lesions, had a sensitivity of 42.3%, specificity of 60.1%, and accuracy of 55.5%. With reference to the dermoscopic findings, it was observed that the greatest specificities for CM ≤6mm were white colour, bluish-grey veil, chrysalis structure and inverted network, whereas the major sensitivities were for brown colour, structureless area, atypical asymmetric pigment network and asymmetric points. Abbasi *et al*. [32] revisited ABCD in 2004, suggesting the addition of the letter “E” with the sense of evolution, summarizing change, growth and evolution. Abbasi *et al*. [7] also reported, in 2008, that small lesions may only have one of the ABC criteria, reinforcing that the follow-up and observation of modification of the lesion may lead to the diagnosis of early cutaneous melanomas. Skvara *et al*. [9] suggested the digital follow-up of small and non-expressive lesions before their immediate removal. They revealed the limitation of dermoscopy in the diagnosis of very early and inexpressive melanomas. For some authors, when we remove suspicious small lesions, we would be preventing these lesions from progressing to unmistakable melanomas [22]. As stated in the study by Drudge *et al*. [33], some early diagnoses were possible because many of these high risk patients had total body photos and new lesions were found by comparing the images and checking all of the pigmented lesions with dermoscopy.

The current study was able to identify two independent dermatoscopic structures associated with the diagnosis of MC ≤ 6mm: streaks (aOR 2.5, 95% CI 1.3 to 4.7, p = 0.006) and structureless area (aOR 2.2, 95% CI 1.2 to 4.0, p = 0.011). It was also the first to identify the presence of a symmetrical atypical pigment network as a criterion that is inversely proportional to the risk of diagnosis of MC ≤ 6mm (aOR 0.4 (95% CI, 0.7 to 0.9). Multiple analysis was adjusted by the following variables: age (continuous), gender, study site, and limb localization. Seidenari *et al*. compared 79 lesions ≤ 6mm with melanomas of larger diameters and found a more atypical pigment network and irregular pigmentation in small melanomas [16].

The present study revealed a great frequency of melanomas (25.6% of lesions) without the “D” of clinical ABCD, i.e. ≤ 6mm in diameter. However, the clinical and dermatoscopic criteria for small lesions did not present high enough sensitivity, specificity and accuracy to suggest that “D” from ABCDE is not useful. Harrington *et al*. [2] in a systematic revision stated that the definition of criteria for the mass autodetection of melanomas is obtained by means of high sensitivity scores and, whenever possible, of high specificity, as established in the studies of the ABCD criterion, which have favored its perpetuation since 1985. Although the existence of microinvasive melanomas is becoming less and less rare, an unrestrained practice of excisions of small lesions without established specific criteria should not be encouraged. Criteria should be carefully considered and combined. These initial melanomas may be incompletely developed melanomas [34]. Woltsche *et al*., in a review, reanalysed melanocytic lesions and reaffirmed that there are lesions that cannot be classified using the clinical, dermatoscopic and histopathological criteria; such lesions would be termed atypical melanocytic proliferations of uncertain significance [35]. In this study, atypical melanocytic nevi were identified in 45.1% of the lesions. Braun-Falco *et al*. studied 261 nevi < 4mm found architectural disorder and atypia in 72% of cases [36]. They suggest that even small lesions should be categorized as atypical nevi due to their histopathological characteristics. They postulate that the histopathological criteria formulated for atypical nevi should be the same as those used for lesions of any size. The dermatoscopic evaluation showed higher sensitivity, while the clinical evaluation was more specific, with this specificity increased by the association of both.

### Limitations of the study

The study has limitations that ought to be pointed out. First, using data from two centers with different *modus operandi* implied the inclusion of patients with different socioeconomic backgrounds. However, this choice may be considered a positive point as it allows the extrapolation of results both for patients using the public service as the private clinic. Another limitation refers to the fact that all patients were evaluated by the same dermatologist, without agreement by different observers. On the other hand, this also allowed the application of uniform criteria for all cases. Besides that, the present study did not employ all of the current dermatoscopic algorithms and checklists, a fact that can imply a limitation of the factors included in the analysis. Finally, the review of all histopathological reports by the same pathologist did not occur, despite all of those involved (both in public hospitals and in private clinics) being dermatopathologists with extensive experience in the diagnosis of melanocytic lesions.

## Conclusion

The diagnosis of small melanocytic lesions assisted by dermoscopy in the detection of melanoma should be encouraged, however with caution, in order to avoid the excessive removal of melanocytic nevi or incipient lesions of still inconclusive diagnoses. Since dermatoscopic criteria for high suspicion regarding melanoma will not always be present in those lesions, dermatoscopic monitoring can help to define the best time to remove them. The clinical ABCDE is to be maintained in association with dermoscopic analysis.

## Supporting information

S1 Table Demographic and epidemiological data of 481 lesions ≤ 6mm in diameter. (DOCX)

S2 Table Clinical characteristics of 481 melanocytic lesions ≤ 6mm in diameter. (DOCX)

S1 File Campos-do-Carmo melanoma plos 2021. (SAV)

## Abreviations

CM: Cutaneous Melanoma
INCA: National Institute of Cancer–Rio de Janeiro–Brazil
S: Sensitivity
E: Specificity
PPV: Positive Predictive Value
NPV: Negative Predictive Value
H&E: Hematoxylin & Eosin

## References

[1] American Cancer Society. Cancer facts & figures 2018. Atlanta, GA: American Cancer Society. 2018. https://www.cancer.org/cancer/melanoma-skin-cancer/about/key-statistics.html. Accessed 4 Nov 2018.

[2] Harrington E, Clyne B, Wesseling N, Sandhu H, Armstrong L, Bennett H, et al. Diagnosing malignant melanoma in ambulatory care: a systematic review of clinical prediction rules. BMJ Open. 2017;7:e014096. doi: 10.1136/bmjopen-2016-014096 28264830 PMC5353325

[3] Benellii C, Roscetti E, Dal Pozzo V. The dermoscopic (7FFM) versus the clinical (ABCDE) diagnosis of small diameter melanoma. Eur J Dermatol. 2000;10:282–7. 10846255

[4] Pizzichetta MA, Argenziano G, Talamini R, Piccolo D, Gatti A, Trevisan G, et al. Dermoscopic criteria for melanoma in situ are similar to those for early invasive melanoma. Cancer. 2001;91:992–7. 11251951

[5] Grichnik JM. Difficult early melanomas. Dermatol Clin. 2001;19:319–25. doi: 10.1016/s0733-8635(05)70269-2 11556240

[6] Ferrara G, Argenziano G, Soyer HP, Corona R, Sera F, Brunetti B, et al. Dermoscopic and histopathologic diagnosis of equivocal melanocytic skin lesions: an interdisciplinary study on 107 cases. Cancer. 2002;95:1094–100. doi: 10.1002/cncr.10768 12209696

[7] Abbasi NR, Yancovitz M, Gutkowicz-Krusin D, Panageas KS, Mihm MC, Googe P, et al. Utility of lesion diameter in the clinical diagnosis of cutaneous melanoma. Arch Dermatol. 2008;144:469–74. doi: 10.1001/archderm.144.4.469 18427040

[8] Bono A, Bartoli C, Baldi M, Moglia D, Tomatis S, Tragni G, et al. Micro-melanoma detection. A clinical study on 22 cases of melanoma with a diameter equal to or less than 3 mm. Tumori. 2004;90:128–31. 15143985 10.1177/030089160409000125

[9] Skvara H, Teban L, Fiebiger M, Binder M, Kittler H. Limitations of dermoscopy in the recognition of melanoma. Arch Dermatol. 2005;141:155–60. doi: 10.1001/archderm.141.2.155 15724011

[10] Bono A, Tolomio E, Trincone S, Bartoli C, Tomatis S, Carbone A, et al. Micro-melanoma detection: a clinical study on 206 consecutive cases of pigmented skin lesions with a diameter < or = 3 mm. Br J Dermatol. 2006;155:570–3. doi: 10.1111/j.1365-2133.2006.07396.x 16911283

[11] Bono A, Tolomio E, Bartoli C, Carbone A, Tomatis S, Zurrida S, et al. Metamorphosis of melanoma. Trends in size and thickness of cutaneous melanoma over one decade at the Istituto Nazionale Tumori, Milan. Tumori. 2008;94:11–3. 18468328 10.1177/030089160809400103

[12] Rigel DS, Russak J, Friedman R. The evolution of melanoma diagnosis: 25 years beyond the ABCDs. CA Cancer J Clin. 2010;60:301–16. doi: 10.3322/caac.20074 20671054

[13] Bono A, Tolomio E, Carbone A, Moglia D, Crippa F, Tomatis S, et al. Small nodular melanoma: the beginning of a life-threatening lesion. A clinical study on 11 cases. Tumori. 2011;97:35–8. 21528661 10.1177/030089161109700107

[14] de Giorgi V, Savarese I, Rossari S, Gori A, Grazzini M, Crocetti E, et al. Features of small melanocytic lesions: does small mean benign? A clinical-dermoscopic study. Melanoma Res. 2012;22:252–6. doi: 10.1097/CMR.0b013e3283527430 22430838

[15] Pupelli G, Longo C, Veneziano L, Cesinaro AM, Ferrara G, Piana S, et al. Small-diameter melanocytic lesions: morphological analysis by means of *in vivo* confocal microscopy. Br J Dermatol. 2013;168:1027–33. doi: 10.1111/bjd.12212 23301553

[16] Seidenari S, Ferrari C, Borsari S, Fabiano A, Bassoli S, Giusti F, et al. Dermoscopy of small melanomas: just miniaturized dermoscopy? Br J Dermatol. 2014;171:1006–13. doi: 10.1111/bjd.12542 23909951

[17] Kamino H, Kiryu H, Ratech H. Small malignant melanomas: clinicopathologic correlation and DNA ploidy analysis. J Am Acad Dermatol. 1990;22:1032–8. doi: 10.1016/0190-9622(90)70147-a 2370327

[18] Gonzalez A, West AJ, Pitha JV, Taira JW. Small-diameter invasive melanomas: clinical and pathologic characteristics. J Cutan Pathol. 1996;23:126–32. doi: 10.1111/j.1600-0560.1996.tb01285.x 8721446

[19] Bono A, Tomatis S, Bartoli C, Tragni G, Radaelli G, Maurichi A, et al. The ABCD system of melanoma detection: a spectrophotometric analysis of the asymmetry, border, color, and dimension. Cancer. 1999;85:72–7. doi: 10.1002/(sici)1097-0142(19990101)85:1&lt;72::aid-cncr10&gt;3.0.co;2-q 9921976

[20] Bono A, Bartoli C, Moglia D, Maurichi A, Camerini T, Grassi G, et al. Small melanomas: a clinical study on 270 consecutive cases of cutaneous melanoma. Melanoma Res. 1999;9:583–6. 10661769

[21] Fernandez EM, Helm KF. The diameter of melanomas. Dermatol Surg. 2004;30:1219–22. doi: 10.1111/j.1524-4725.2004.30379.x 15355364

[22] Goldsmith SM, Solomon AR. A series of melanomas smaller than 4 mm and implications for the ABCDE rule. J Eur Acad Dermatol Venereol. 2007;21:929–34. doi: 10.1111/j.1468-3083.2006.02115.x 17659002

[23] Helsing P, Loeb M. Small diameter melanoma: a follow-up of the Norwegian Melanoma project. Br J Dermatol. 2004;151:1081–3. doi: 10.1111/j.1365-2133.2004.06248.x 15541089

[24] Filho RSO, Oliveira DA, Souza MC, Silva M, Brandao MD. Suspected melanoma only when the lesion is greater than 6mm may harm patients. Einstein (Sao Paulo). 2015;13:506–9. 26761547 10.1590/S1679-45082015AO3436 PMC4878622

[25] Friedman RJ, Rigel DS, Kopf AW. Early detection of malignant melanoma: the role of physician examination and self-examination of the skin. CA Cancer J Clin. 1985;35(3):130–51. doi: 10.3322/canjclin.35.3.130 3921200

[26] Blum A, Rassner G, Garbe C. Modified ABC-point list of dermoscopy: A simplified and highly accurate dermoscopic algorithm for the diagnosis of cutaneous melanocytic lesions. J Am Acad Dermatol. 2003;48:672–8. doi: 10.1067/mjd.2003.282 12734495

[27] Carli P, De Giorgi V, Chiarugi A, Nardini P, Mannone F, Stante M, et al. Effect of lesion size on the diagnostic performance of dermoscopy in melanoma detection. Dermatology. 2003;206:292–6. doi: 10.1159/000069939 12771468

[28] Salerni G, Alonso C, Fernández-Bussy R. A series of small-diameter melanomas on the legs: dermoscopic clues for early recognition. Dermatol Pract Concept. 2015;5:31–6. doi: 10.5826/dpc.0504a08 26693087 PMC4667599

[29] Annessi G, Bono R, Sampogna F, Faraggiana T, Abeni D. Sensitivity, specificity, and diagnostic accuracy of three dermoscopic algorithmic methods in the diagnosis of doubtful melanocytic lesions: the importance of light brown structureless areas in differentiating atypical melanocytic nevi from thin melanomas. J Am Acad Dermatol. 2007;56:759–67. doi: 10.1016/j.jaad.2007.01.014 17316894

[30] Lallas A, Longo C, Manfredini M, Benati E, Babino G, Chinazzo C, et al. Accuracy of dermoscopic criteria for the diagnosis of melanoma in situ. JAMA Dermatol. 2018;154:414–9. doi: 10.1001/jamadermatol.2017.6447 29466542 PMC5876885

[31] Tognetti L, Cevenini G, Moscarella E, et al. Validation of an integrated dermoscopic scoring method in an European teledermoscopy web platform: the iDScore project for early detection of melanoma. J Eur Acad Dermatol Venereol. 2020;34(3):640–647. doi: 10.1111/jdv.15923 31465600

[32] Abbasi NR, Shaw HM, Rigel DS, Friedman RJ, McCarthy WH, Osman I, et al. Early diagnosis of cutaneous melanoma: revisiting the ABCD criteria. JAMA. 2004;292:2771–6. doi: 10.1001/jama.292.22.2771 15585738

[33] Drugge ED, Volpicelli ER, Sarac RM, Strang SR, Elston DM, Drugge RJ. Micromelanomas identified with time-lapse total body photography and dermoscopy. J Am Acad Dermatol. 2018;78:182–3. doi: 10.1016/j.jaad.2017.07.049 29241777

[34] Quintella DC, Campos-do-Carmo G, Quintella LP, Cuzzi T. Diagnóstico histopatológico de lesões melanocíticas pequenas suspeitas de melanoma. Anais Brasileiros de Dermatologia. 2017;92(3):379–83. doi: 10.1590/abd1806-4841.20175996 29186251 PMC5514579

[35] Woltsche N, Schmid-Zalaudek K, Deinlein T, Rammel K, Hofmann-Wellenhof R, Zalaudek I. Abundance of the benign melanocytic universe: Dermoscopic-histopathological correlation in nevi. J Dermatol. 2017;44(5):499–506. doi: 10.1111/1346-8138.13808 28447347

[36] Braun-Falco M, Hein R, Ring J, McNutt N S. Histopathological characteristics of small diameter melanocytic naevi. J Clin Pathol. 2003; 56:459–464. doi: 10.1136/jcp.56.6.459 12783974 PMC1769983

