# Supplementary material for: Melanocytic lesions ≤ 6mm: Prospective series of 481 melanocytic trunk and limb lesions in Brazil
Source: PLoS One. 2021 Jun 8;16(6):e0252162. doi: 10.1371/journal.pone.0252162 (PMC8186794; doi:10.1371/journal.pone.0252162)
Supplement: S1 Table — (DOCX) [file pone.0252162.s001.docx]

**S1 Table.** **Demographic and epidemiological data of 481 lesions ≤ 6mm in diameter**

| **Variables** | **CM**  **N (%)** | **Non-CM**  **N (%)** | **p** |
| --- | --- | --- | --- |
| Institution |  |  | **< 0.001** |
| Public Service | 33 (26.8) | 193 (53.9) |  |
| Private Clinic | 90 (73.2) | 165 (46.1) |  |
| Sex |  |  | 0.809 |
| Female | 72 (58.5) | 214 (59.8) |  |
| Male | 51 (41.5) | 144 (40.2) |  |
| Age (years ) |  |  | **0.007** |
| ≤30 | 6 (4.9) | 50 (14.0) |  |
| 31-50 | 54 (43.9) | 167 (46.6) |  |
| 51-70 | 45 (36.6) | 114 (31.8) |  |
| >70 | 18 (14.6) | 27 (7.5) |  |
| Phototype |  |  | 0.685 |
| I | 14 (11.4) | 39 (10.9) |  |
| II | 72 (58.5) | 213 (59.5) |  |
| III | 37 (30.1) | 102 (28.5) |  |
| IV | 0 (0.0) | 4 (1.1) |  |
| Hair color |  |  | 0.823 |
| Blond | 22 (17.9) | 78 (21.8) |  |
| Red | 10 (8.1) | 28 (7.8) |  |
| Brown | 80 (65.0) | 219 (61.2) |  |
| Black | 11 (8.9) | 33 (9.2) |  |
| Eye color |  |  | 0.183 |
| Blue | 13 (10.6) | 30 (8.4) |  |
| Green | 19 (15.4) | 86 (24.0) |  |
| Brown | 89 (72.4) | 232 (64.8) |  |
| Black | 2 (1.6) | 10 (2.8) |  |
| Personal CM history |  |  | 0.899 |
| Yes | 60 (48.8) | 177 (49.4) |  |
| No | 63 (51.2) | 181 (50.6) |  |
| Multiple primary melanomas |  |  | 0.453 |
| Yes | 19 (15.4) | 66 (18.4) |  |
| No | 104 (84.6) | 292 (81.6) |  |
| History of CM metastases |  |  | 0.189 |
| Yes | 3 (2.4) | 19 (5.3) |  |
| No | 120 (97.6) | 339 (94.7) |  |
| Family history melanoma |  |  | **0.022** |
| Yes | 46 (37.4) | 95 (26.5) |  |
| No | 77 (62.6) | 263 (73.5) |  |
| Presence of common nevi |  |  | 0.435 |
| Yes ≤ 50 | 18 (14.6) | 39 (10.9) |  |
| Yes from 51 to 100 | 22 (17.9) | 77 (21.5) |  |
| Yes > 100 | 83 (67.5) | 242 (67.6) |  |
| Presence of atypical nevi* |  |  | 0.147 |
| Yes ≤ 50 | 29 (23.6) | 116 (32.4) |  |
| Yes > 50 | 65 (52.8) | 176 (49.2) |  |
| None | 29 (23.6) | 66 (18.4) |  |
| Sunburn** |  |  | 0.847 |
| Yes, First degree | 60 (48.8) | 164 (45.8) |  |
| Yes, ≥ Second degree | 52 (42.3) | 161 (45.0) |  |
| None | 11 (8.9) | 33 (9.2) |  |
| History of skin carcinoma |  |  | 0.626 |
| Yes | 40 (32.5) | 108 (30.2) |  |
| No | 83 (67.5) | 250 (69.8) |  |

Statistically significant p values are shown in bold type. CM = Cutaneous Melanoma. Non-CM = benign melanocytic lesion or an atypical nevus.

* Atypical nevi – nevi with architectural disorder or cellular atypia

** Sunburn – redness (first degree) and blisters (second degree) after sun exposure
