# Supplementary material for: Melanocytic lesions ≤ 6mm: Prospective series of 481 melanocytic trunk and limb lesions in Brazil
Source: PLoS One. 2021 Jun 8;16(6):e0252162. doi: 10.1371/journal.pone.0252162 (PMC8186794; doi:10.1371/journal.pone.0252162)
Supplement: S2 Table — (DOCX) [file pone.0252162.s002.docx]

**S2 Table.** **Clinical characteristics of 481 melanocytic lesions ≤ 6mm in diameter**

| **Variables** | **CM**  **N (%)** | **Non-CM**  **N (%)** | **p** |
| --- | --- | --- | --- |
| A – Asymmetry |  |  | 0.632 |
| Yes | 57 (46.3) | 157 (43.9) |  |
| No | 66 (53.7) | 201 (56.1) |  |
| B – Asymmetric borders |  |  | 0.553 |
| Yes | 56 (45.5) | 152 (42.5) |  |
| No | 67 (54.5) | 206 (57.5) |  |
| C – Colors |  |  | 0.596 |
| More than one | 49 (39.8) | 133 (37.2) |  |
| Single color | 74 (60.2) | 225 (62.8) |  |
| D – Size in millimeters |  |  | 0.154 |
| 1 | 3 (2.4) | 8 (2.2) |  |
| 2 | 28 (22.8) | 54 (15.1) |  |
| 3 | 31 (25.2) | 108 (30.2) |  |
| 4 | 31 (25.2) | 92 (25.7) |  |
| 5 | 16 (13.0) | 70 (19.6) |  |
| 6 | 14 (11.4) | 26 (7.3) |  |
| E – Evolution |  |  | 0.116 |
| Unknown | 70 (56.9) | 194 (54.2) |  |
| New | 17 (13.8) | 39 (10.9) |  |
| Old | 1 (0.8) | 21 (5.9) |  |
| Altered | 35 (28.5) | 104 (29.1) |  |
| Elementary lesion |  |  | **0.005** |
| Macula* | 107 (87.0) | 268 (74.9) |  |
| Papule | 16 (13.0) | 90 (25.1) |  |
| Location |  |  | **<0.001** |
| Trunk | 43 (35.0) | 224 (62.6) |  |
| Limbs | 80 (65.0) | 134 (37.4) |  |

Statistically significant p values are shown in bold type. CM = Cutaneous Melanoma

* Macula - a flat, non-papular lesion
